# Tools and Databases of the KOMICS Web Portal for Preprocessing, Mining, and Dissemination of Metabolomics Data

**DOI:** 10.1155/2014/194812

**Published:** 2014-04-09

**Authors:** Nozomu Sakurai, Takeshi Ara, Mitsuo Enomoto, Takeshi Motegi, Yoshihiko Morishita, Atsushi Kurabayashi, Yoko Iijima, Yoshiyuki Ogata, Daisuke Nakajima, Hideyuki Suzuki, Daisuke Shibata

**Affiliations:** ^1^Kazusa DNA Research Institute, 2-6-7 Kazusa-kamatari, Kisarazu, Chiba 292-0818, Japan; ^2^JST, National Bioscience Database Center (NBDC), 5-3 Yonbancho, Chiyoda-ku, Tokyo 102-0081, Japan; ^3^Department of Nutrition and Life Science, Kanagawa Institute of Technology, 1030 Shimo-ogino, Atsugi, Kanagawa 243-0292, Japan; ^4^Graduate School of Life and Environmental Sciences, Osaka Prefecture University, Sakai, Osaka 599-8531, Japan

## Abstract

A metabolome—the collection of comprehensive quantitative data on metabolites in an organism—has been increasingly utilized for applications such as data-intensive systems biology, disease diagnostics, biomarker discovery, and assessment of food quality. A considerable number of tools and databases have been developed to date for the analysis of data generated by various combinations of chromatography and mass spectrometry. We report here a web portal named KOMICS (The Kazusa Metabolomics Portal), where the tools and databases that we developed are available for free to academic users. KOMICS includes the tools and databases for preprocessing, mining, visualization, and publication of metabolomics data. Improvements in the annotation of unknown metabolites and dissemination of comprehensive metabolomic data are the primary aims behind the development of this portal. For this purpose, PowerGet and FragmentAlign include a manual curation function for the results of metabolite feature alignments. A metadata-specific wiki-based database, Metabolonote, functions as a hub of web resources related to the submitters' work. This feature is expected to increase citation of the submitters' work, thereby promoting data publication. As an example of the practical use of KOMICS, a workflow for a study on *Jatropha curcas* is presented. The tools and databases available at KOMICS should contribute to enhanced production, interpretation, and utilization of metabolomic Big Data.

## 1. Introduction

A metabolome, which comprises comprehensive data on quantification of metabolites in an organism calculated using metabolomic technologies [9, 10], has been increasingly used for the analysis and practical applications of biological and environmental systems. Within the data-intensive systems biology discipline, metabolomics is particularly important compared to other “omics” (genome, transcriptome, and proteome) disciplines since metabolomes are more closely related to phenotype and regulate gene and protein expression networks [11–13]. Mass spectrometry (MS) and nuclear magnetic resonance spectroscopy (NMR) are complementary techniques often used for the detection and identification of metabolites. MS technology has integrated separation techniques and is used in most cases because of its sensitivity, selectivity, speed, and broad applicability [14–16]. Owing to the wide range of chemical diversity, there is no ideal apparatus that is capable of analyzing all possible metabolites. Combinations of separation techniques with MS, such as liquid chromatography- (LC-) MS, gas chromatography- (GC-) MS, and capillary electrophoresis- (CE-) MS, are chosen according to a study's purpose [17–19]. Metabolomics technology, including instrumental analysis, detection and identification of metabolites, statistical interpretation, and generation of hypotheses with computational support, is used for a variety of studies, such as functional analysis of biological systems [20–22], biomarker discovery [23, 24], medical diagnostics [14, 25], quality assessment of foods [26, 27], evaluation of genetically modified crops [28, 29], and assessment of environmental pollution [30, 31].

**Figure 1:**
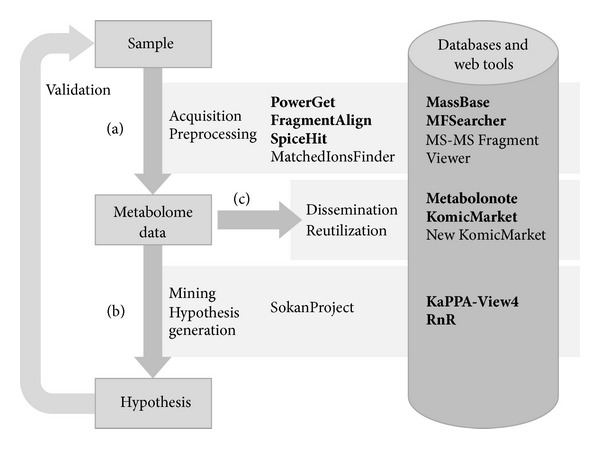
A typical workflow of a metabolomics study and KOMICS-relevant tools and databases. The process of data acquisition and preprocessing (a) is required for generating the metabolome data. A working hypothesis is generated by interpreting the metabolome data (b), and the cycle is completed after validating the hypotheses by further analyses (the arrow on the left side). The metabolome data are published in the databases (c) and utilized for preprocessing and data mining. The tools and databases introduced in the main text of this paper are shown in bold face.

A considerable number of software tools and databases have been developed for processing the complicated and multidimensional metabolome datasets generated by various types of MS-based instruments [32–35]. A typical workflow of metabolomic data analysis includes the following processes: (a) preprocessing of raw data for extraction of metabolite features, annotation of the metabolites, and finally generation of metabolome data; (b) mining and visualization of metabolome data for statistical interpretation of its nature and hypothesis generation; (c) storing and disseminating the data for further utilization and comparison (Figure 1). XCMS2 [36], MzMine2 [37], MathDAMP [38], MetAlign [39], and MET-IDEA [40] are typical tools for preprocessing including detection, alignment, and annotation of metabolite features. Some of these tools also provide statistical analysis functions for data interpretation. MassBank [41], METLIN [42], PRIMe [43], and HMDB [44] are available as references of mass spectra for metabolite annotation. The metabolite data are interpreted by means of the genome information from compound databases such as KNApSAcK [45], PubChem [46], and Chemspider (http://www.chemspider.com/) and by means of metabolic pathway databases including KEGG [47], BioCyc [48], and Reactome [49], which enable data visualization on pathway maps. The raw and processed data are stored publicly in databases such as PlantMetabolomics.org [50], GMD@CSB.DB [51], SetupX (currently not available), MetabolomeExpress [52], MetaboLights [53], and Metabolomics Workbench (http://www.metabolomicsworkbench.org/).

We report here a portal website named* KOMICS* (The Kazusa Metabolomics Portal, http://www.kazusa.or.jp/komics/), which hosts tools and databases that we developed for metabolomics. Although an increasing number of tools and databases have become available, two major issues remain to be resolved, that is, comprehensiveness of metabolites [54, 55] and data dissemination [53, 56, 57]. Our primary aim in developing data preprocessing tools is to help researchers with the manual annotation process that remains essential for nontarget metabolomics [54]. PowerGet for LC-high-resolution-MS and FragmentAlign for GC-MS are tools that enable curation of peak alignment results. SpiceHit is a high-throughput metabolite identification tool for CE-MS analysis using the selected ion monitoring (SIM) method. We have also developed data mining and visualization tools for the generation of working hypotheses (KaPPA-View and RnR). Real data is indispensable for comparative analysis and for the development and improvement of preprocessing tools [53, 58]. MassBase is one of the largest raw data repositories, and KomicMarket is a database of metabolic profiling data. We developed a metadata-specific database, Metabolonote, to promote data publication by researchers. These resources for a wide range of metabolome data processing are expected to contribute to improved production and utilization of metabolomic data.

## 2. Materials and Methods

The standalone tools for metabolome data production, PowerGet, FragmentAlign, and SpiceHit, were developed in Java (Oracle Corporation). The web-based tools and databases were developed and are run in Apache, PHP, Perl, MySQL, Java, and Tomcat on Linux servers. The KOMICS website was constructed using the content management system “Joomla!” running on a Linux server with Apache, PHP, and MySQL. The details of the development and license information are described in the individual introduction pages of KOMICS, in manuals, or in other relevant help resources. The tools and databases are freely available to academic users.

Details of the analytical methods for the evaluation of preprocessing tools are described in the Supplementary Material (see Supplementary Material available online at http://dx.doi.org/10.1155/2014/194812).

## 3. Results and Discussion

The tools and databases we have developed and provided at the KOMICS web portal are classified into three categories according to the typical workflow of metabolomic data analysis, namely, (a) preprocessing tools, (b) data mining tools, and (c) databases for data dissemination (Figure 1). Here we describe several representative examples. All the currently available tools and databases are listed in Table 1. The number of records in each metabolomics-related database is shown in Table 2. The formats of input and output files and the availability of sample datasets are summarized in Table 3.

### 3.1. Data Preprocessing Tools

#### 3.1.1. PowerGet

PowerGet is a standalone Java software package for detection, alignment, and annotation of metabolite features from data obtained using LC-high-resolution-MS (HRMS). Accurate mass values measured by HRMS, such as Fourier transform ion cyclotron resonance MS and Orbitrap MS (Thermo Fisher), allow users to predict the elemental composition of a metabolite. The intensity ratio of ^13^C to ^12^C isotopic ion peaks is useful for estimating the number of carbon atoms in a molecule. Estimation of ion adducts attached to the metabolites by coeluted ions is helpful for calculating elemental composition and for search of compound databases by mass values of nonionized molecules. The PowerFT module in PowerGet attaches these data automatically to all metabolite features in the LC-HRMS data. In the PowerMatch module, the metabolite features are aligned among the samples taking into account the similarity of MS/MS fragmentation patterns. A tool for refining the alignment results, MatchedIonsFinder [1], is also available via KOMICS.

To evaluate the accuracy of mass values of the peaks detected using PowerGet, the mass differences between a theoretical mass and a detected mass were compared to those of the peaks detected using the commercial software, Xcalibur (see Supplementary Method S1). PowerGet exhibited greater accuracy (0.579 ± 0.481 ppm (mean ± SD)) than Xcalibur (0.783 ± 0.563 ppm) in the evaluation of 143 standard compounds (Supplementary Table S1).

**Figure 2:**
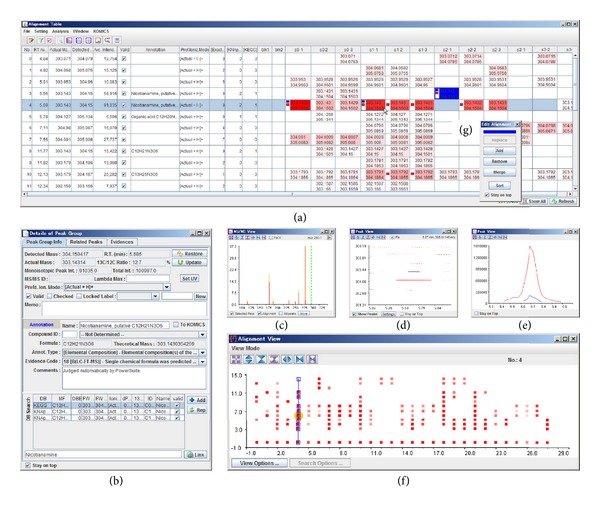
The alignment-editing function of the PowerMatch module of PowerGet. (a) The Alignment Table shows the alignment results of the peaks detected in each sample. The intensity of peaks is summarized in another window (f). The details of the peak information (b), MS/MS fragments (c), appearance of peripheral peaks (d), and peak shape (e) are shown for the user-selected peaks. A misaligned peak (the blue colored cell in panel a) can be merged to an appropriate row using the Edit Alignment function (g), by immediately checking the detailed information for the peak (b–e).

One of the unique functions of PowerGet is that the alignment results are manually editable: a user can promptly check metabolite's characteristics, such as mass chromatogram shape, existence of adjacent features, and MS/MS fragmentation patterns, by means of a graphical user interface (GUI), as shown in Figure 2. Alignment is essential for preparing matrices of samples to metabolite intensity data for further comparison and statistical analysis. Alignment is highly valuable when users need to annotate the metabolites, especially for unknown features. By comparing the features from several replicate samples, (1) the estimation error of the ion adducts is verified, (2) accuracy of mass measurement can be improved, and (3) reproducibly detected features are prioritized for further annotation. Therefore, alignment errors should be assessed and corrected during detailed annotation of unknown metabolites. PowerGet is utilized in preparing data for KomicMarket and Bio-MassBank (http://bio.massbank.jp/ ).

#### 3.1.2. FragmentAlign

**Figure 3:**
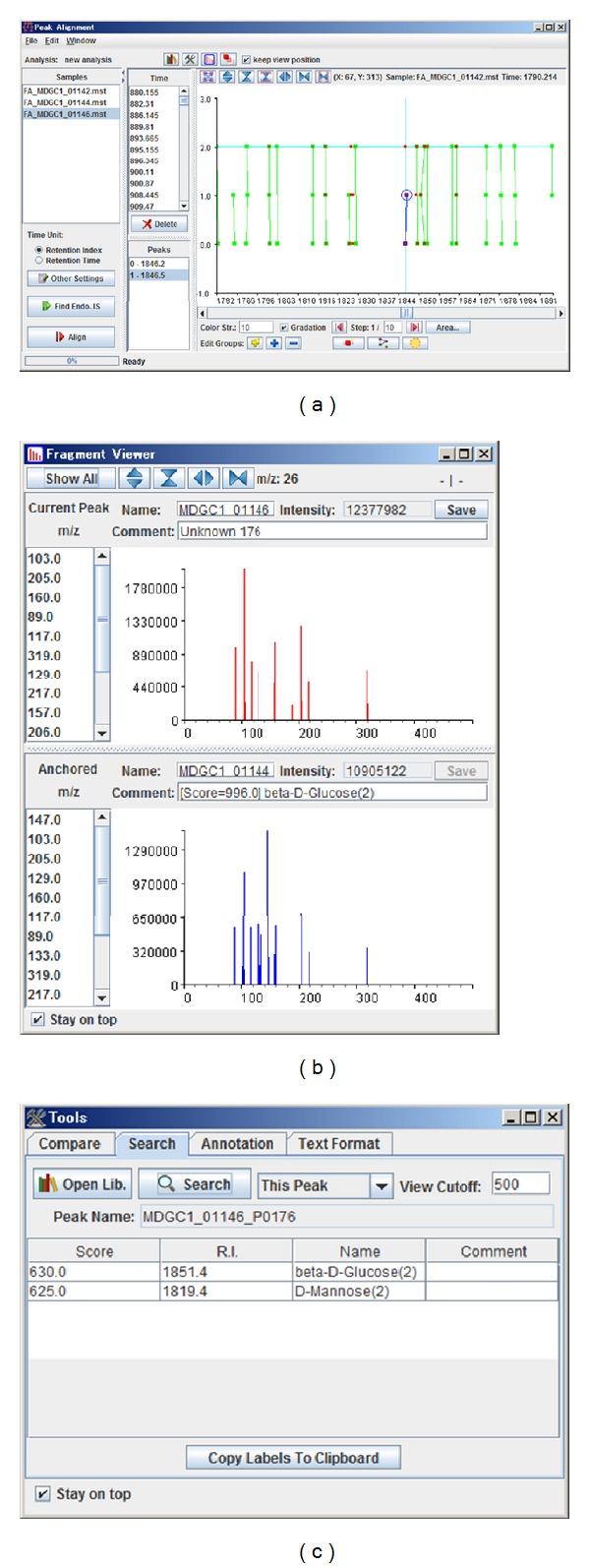
Screenshots of FragmentAlign. An alignment result from 3 samples is depicted (a). The electron ionization (EI) mode of a fragmentation pattern of the metabolite peak is presented in the Fragment Viewer panel (b). The metabolites are annotated by comparing the similarity of the fragment patterns to those obtained from standard compounds (c).

This is a standalone Java tool designed for GC-MS data analysis with functions for alignment and annotation of metabolite features. A GUI for editing the alignment results is also implemented in this software (Figure 3). The similarity of fragment ion patterns generated by electron ionization (EI) is taken into account in the alignment of metabolite features. The metabolite features can also be annotated based on EI fragment patterns, by comparing to patterns from standard compounds. The fragment pattern data of standard compounds can be imported and utilized when the data is written in the format defined by the National Institute of Standards and Technology (NIST), USA.

To evaluate the applicability of data matrices generated by FragmentAlign for further statistical analyses, a principal component analysis (PCA) was conducted using the GC-MS data obtained from 3 biological sources:* Arabidopsis* leaves,* Lotus japonicus* leaves, and* Arabidopsis* cultured cells. Five replicates of each source were mapped to similar positions, whereas the 3 sources were mapped separately from one another on the score plot (Supplementary Figure S1). High-correlation coefficients for peak intensity within the replicates were observed (Supplementary Figure S2). These results suggest that appropriate feature extraction and generation of data matrices can be performed successfully using FragmentAlign.

#### 3.1.3. SpiceHit

The standalone Java tool SpiceHit is intended for high-throughput identification of metabolite features detected using the selected ion monitoring (SIM) method in CE-MS. The metabolite features are identified based on migration times relative to internal standard compounds and are compared to those of the standard compound library prepared in-house. The tool is designed for processing a large number of data files in a high-throughput manner; it requires checking and correcting the assignment errors manually.

To ascertain whether SpiceHit is applicable to practical data analysis, the accuracy of peak quantification was compared to that acquired using the commercial software ChemStation (Agilent Technologies, Palo Alto, CA). In the detection of amino acids, the results from SpiceHit were strongly correlated with those from ChemStation, as well as with theoretical concentrations (Supplementary Table S4). Similar relative standard deviation (RSD) values for each amino acid in triplicate analyses were observed for SpiceHit and ChemStation (Supplementary Figure S3). Good linearity of peak areas common to SpiceHit and ChemStation was observed in the amino acid peaks detected in the biological samples (Supplementary Figure S4). These results suggest that the accuracy and the sensitivity of peak quantification by SpiceHit are similar to those of ChemStation and that SpiceHit is suitable for practical use.

#### 3.1.4. MFSearcher

This is a web service that allows for rapid prediction of elemental composition from accurate mass values and for rapid searching of compound databases [3]. A GUI tool for MFSearcher queries is also provided as a module in PowerGet. PowerGet has a batch search function for querying thousands of detected metabolite features via MFSearcher.

Because MFSearcher is a RESTful web service, the query parameters for MFSearcher should be included in the description of a URL. Numerous sample queries are available as URL links on the MFSearcher website.

### 3.2. Data Mining Tools

#### 3.2.1. KaPPA-View

This is a web-based tool for the visualization of metabolomic data on metabolic pathway maps from the Kyoto Encyclopedia of Genes and Genomes (KEGG) [5]. The degree of change in metabolite abundance between several samples is expressed as hue of the compound symbols drawn on the KEGG pathway maps, based on the compound IDs assigned. Alterations in transcriptome data can be simultaneously depicted on the maps. This tool can be used for the integrated analysis of metabolomic, transcriptomic, and possibly proteomic data.

Sample data for testing the color representations on the pathway maps are available on the “Analysis” page of KaPPA-View. Users can select the items according to the directions presented on the page. Sample files for input data are available on the “Download” page.

#### 3.2.2. RnR

This database contains data on the relationship between metabolites and genes; these data were generated via simultaneous measurement of the metabolome and transcriptome of approximately 200 transgenic cultured cell lines of the model plant* Arabidopsis*. The gene expression patterns and metabolite changes resulting from specific transgenes are compiled in the database. Users can search, for example, genes that can affect the abundance of the queried metabolites and vice versa. The database should contribute to knowledge discovery related to gene-to-metabolite regulatory networks in* Arabidopsis* cells.

To view an example dataset, a clickable pie chart of metabolites is presented on the main page of the RnR website. Clicking on a section of the pie chart will show a list of metabolites. After choosing a metabolite name, users will be able to view candidate genes that are related to the metabolite.

### 3.3. Databases for Data Dissemination

#### 3.3.1. MassBase

The primary purpose of MassBase is the distribution of raw data generated by analytical instruments. Dissemination of raw data would enable the development and improvement of data analysis tools by bioinformatics researchers [53]. Binary raw data and near-raw text data exported from raw binary results are provided.

**Figure 4:**
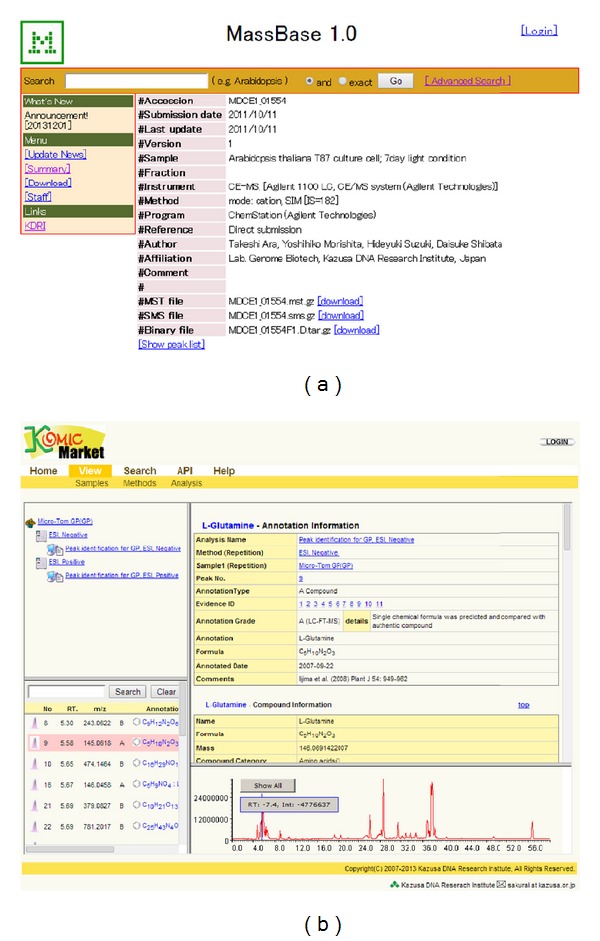
Screenshots of the web interface of MassBase (a) and KomicMarket (b).

Users can search records by sample name, sample description, instrument type, and ionization mode on the “Advanced Search” page (Figure 4(a)). A summary of the records classified by species and instrument type is available on the “Summary” page.

#### 3.3.2. KomicMarket

KomicMarket is a sample-centric database aimed at the distribution of metabolic profiles with and without metabolite annotations (Figure 4(b)). Previous results on the detection and annotation of metabolite features in certain samples can serve as good references for future metabolite annotations [56].

The records can be queried by keywords in the sample descriptions, including peak characteristics such as mass values, and in annotations of the peaks via the GUI on the KomicMarket website. The system provides application programming interfaces (APIs) for performing software-based querying of the data. Using the APIs, we employed the MFSearcher module of PowerGet to search metabolites in KomicMarket.

#### 3.3.3. Metabolonote

This Semantic MediaWiki-based database is intended for managing metadata, which is the detailed information on experimental procedures accompanying the generation of data. Metabolonote is expected to accelerate publication of metabolomics data. The raw data obtained from the experiments or the processed data derived from them are not the target of Metabolonote, and the “actual data” are normally stored in other databases specifically built for a given purpose. Separation of the management of complicated metadata of metabolomics from the management of actual data makes it possible to share the same metadata among multiple actual databases such as raw data repositories, metabolic profile databases, reference libraries of MS/MS, and research papers. One-stop-shop management of complicated metadata of metabolomics eliminates the redundant management of metadata in several databases and reduces labor for data submitters. We defined a simple data format named* Togo metabolomics data format* (TogoMD) for easier description of metadata. Specifications of the TogoMD format are documented on the Metabolonote website (http://metabolonote.kazusa.or.jp/TogoMetabolomeDataFormat). Metabolonote provides application programming interfaces (APIs) for semantic searching of the records and retrieval of metadata. Because Metabolonote is a wiki system, it allows the submitters to attach free additional information about the metadata, such as images of the samples, video recordings of tricky analytical procedures, and links to a journal's website where the results are published. Therefore, metadata written by the submitter function as a hub of the web data resources related to the submitter's work. The increased presence of the submitters' published work on the web should increase the citations by others [58]. Therefore, the wiki system is expected to facilitate the dissemination of data to the public. Metabolonote is already linked to seven actual databases, including MassBase, KomicMarket, Bio-MassBank, and Riken PRIMe.

The metadata deposited in Metabolonote are listed on the “Public Pages.” The registered metadata are semantically searchable by various items (and combinations thereof) on the “Metadata Search” page.

### 3.4. Practical Use

**Figure 5:**
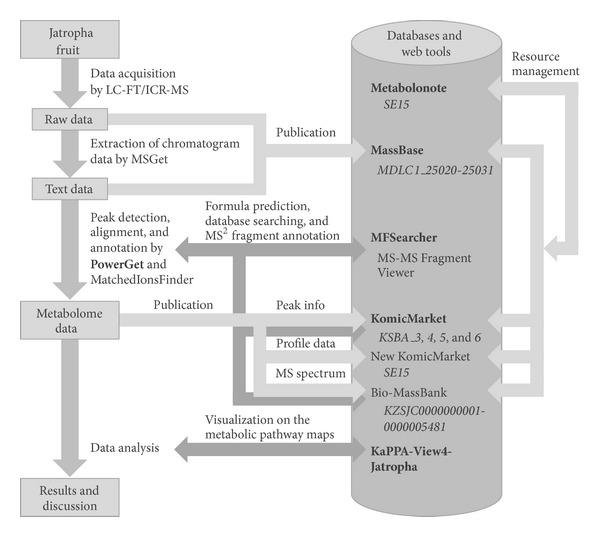
A schematic representation of the workflow for analysis of metabolomic changes in the developing fruit of* Jatropha curcas* L. The tools and databases introduced in the main text are shown in bold face. The accession IDs of the data in each database are shown in italics.

Here we present a workflow for a metabolomics study of* Jatropha curcas* L. [59, 60], a biofuel plant, to illustrate an example of the practical use of the KOMICS resources (Figure 5). LC-Orbitrap-MS analyses were conducted using 4 developmental stages of* J. curcas* fruit samples. The acquired data were primarily recorded as a binary raw file with commercial software (Xcalibur, Thermo Fisher). To analyze the data with PowerGet, the chromatogram data were exported to text files using the MSGet tool, which is also available on the KOMICS website. The raw files and extracted text files were published on MassBase. The text data were then processed using the PowerGet tool to generate the metabolomic data. MatchedIonsFinder was used to refine the alignment results. MFSearcher was used for high-throughput search of elemental composition and compound databases. MS-MS Fragment Viewer was used for interpreting MS/MS fragments in the metabolite annotations. The peak information, profile data (in the TogoMD format), and MS spectrum data were stored on KomicMarket (on the New KomicMarket temporary website) and on Bio-MassBank, respectively. These data were recursively used for metabolite annotations during the preprocessing step. Subsequently, the nature of the metabolomic data was interpreted by visualization on pathway maps using KaPPA-View4 and other statistical analyses. Consequently, a drastic change in metabolites during the maturation of* J. curcas* fruit was detected, and these data should contribute to further analysis of oil production by* J. curcas*. The record in Metabolonote (metadata ID: SE5) is a good guidepost for finding data resources deposited in various databases on the web.

## 4. Conclusions

We have developed various tools and databases for a wide range of processes related to metabolomic studies: preprocessing, data mining, and publication. To our knowledge, PowerGet and FragmentAlign are the first tools to allow users to curate alignment results via GUI. The unique concept of a metadata-specific database should accelerate data publication and dissemination. This infrastructure is expected to assist researchers to attain superior utilization of metabolomics' Big Data. Nonetheless, annotation of novel metabolites (the so-called unknown unknowns) remains a serious bottleneck in building comprehensive metabolomic datasets [16, 54]. Continuous efforts are needed to improve and automate annotation tasks. In addition, a systematic collection of annotation skills from experts will be necessary in the near future, as will the analysis and transfer of these skills to the public domain for education of fledgling annotators.

## Supplementary Material

The Supplementary Material include 3 supplementary methods and 4 supplementary figures as follows.
Supplementary Method S1: The method for the evaluation of the accuracy of mass values of the peaks detected using PowerGet tool.
Supplementary Method S2: The method for the evaluation of applicability of a data matrix resulting from peak alignment in FragmentAlign tool to a comparative metabolomics analysis.
Supplementary Method S3. The method for the evaluation of accuracy of peak area quantified using SpiceHit tools.
Supplementary Figure S1: Principal component analysis (PCA) of metabolite peaks in *Arabidopsis* leaves, *Lotus japonicus* leaves, and *Arabidopsis* cultured cells detected using GC-MS and aligned by means of FragmentAlign.
Supplementary Figure S2: Correlation coefficients of the values of peak intensity aligned in FragmentAlign.
Supplementary Figure S3: Reproducibility of peak area quantification in SpiceHit and ChemStation.
Supplementary Figure S4: Comparison of values of the peak area quantified using SpiceHit and ChemStation.
Four supplementary tables are as follows.
Supplementary Table S1: The standard compounds used for the comparison of accuracy of mass values obtained using PowerGet and Xcalibur.
Supplementary Table S2: The data used for peak alignment of GC-MS data through FragmentAlign.
Supplementary Table S3: The data used for the evaluation of quantification of peaks using SpiceHit.
Supplementary Table S4: Comparison of accuracy of peak area quantification in SpiceHit and in ChemStation.

## Figures and Tables

**Table 1:** The tools and databases available at KOMICS (as of November 2013).

Name	Description	URL	Reference
Standalone tools			
PowerGet	Metabolite detection, alignment, and annotation tool for LC-high-resolution-MS.	http://www.kazusa.or.jp/komics/software/PowerGet	
MatchedIonsFinder	Revising tool for metabolite alignment results from LC-MS analyses.	http://www.kazusa.or.jp/komics/software/MatchedIonsFinder	[1]
FragmentAlign	Metabolite alignment and annotation tool for GC-MS.	http://www.kazusa.or.jp/komics/software/FragmentAlign	
SpiceHit	High-throughput metabolite detection and annotation tool for SIM analysis in CE-MS.	http://www.kazusa.or.jp/komics/software/SpiceHit	
KAGIANA	Microsoft Excel-based tool for exploring the function of *Arabidopsis* genes.	http://webs2.kazusa.or.jp/kagiana/	[2]
SokanProject	A tool for calculating Pearson's correlation coefficients.	http://www.kazusa.or.jp/komics/software/SokanProject	

Web tools			
MFSearcher	Web service for rapid prediction of elemental composition and database searching by accurate mass values.	http://webs2.kazusa.or.jp/mfsearcher/	[3]
DAGViz	Visualization tool for similarities of gene ontology annotations.	http://www.pgb.kazusa.or.jp/dagviz/	[4]

Databases			
MassBase	Largest repository of metabolomics raw data.	http://webs2.kazusa.or.jp/massbase/	
KomicMarket	Sample-centric database for metabolomic profile data.	http://webs2.kazusa.or.jp/komicmarket/	
New KomicMarket temporary website	Developmental version of KomicMarket.	http://webs2.kazusa.or.jp/new_km_tmp/	
KaPPA-View4	Pathway database for visualizing metabolome and transcriptome data.	http://kpv.kazusa.or.jp/	[5]
Metabolonote	Metadata-specific Semantic MediaWiki-based database.	http://metabolonote.kazusa.or.jp/	
MS-MS Fragment Viewer	Database for MS/MS fragmentation data of 115 flavonoids.	http://webs2.kazusa.or.jp/msmsfragmentviewer/	
RnR	Database providing metabolite-to-gene relationships calculated from ~200 transgenic *Arabidopsis* cells.	http://webs2.kazusa.or.jp/kagiana/rnr	
CoP	Gene-to-gene coexpression database for 8 plant species calculated using the Confeito algorithm.	http://webs2.kazusa.or.jp/kagiana/cop0911	[6]
KATANA	Cross-search system for *Arabidopsis* genes.	http://www.kazusa.or.jp/katana/	[7]
ARTRA	Database of probe information of *Arabidopsis* DNA microarray data (developed by Takara Bio Inc.).	http://artra.kazusa.or.jp/artra/ARI3_101/	[8]
FuLoja	Database of *Lotus japonicus* full-length cDNA obtained in the NEDO project.	http://webs2.kazusa.or.jp/IntegrationDBRS/FuLoja/	
PMPj-Blast	Database of ESTs, cDNAs, and oligo DNA microarray probes for *Lotus japonicus* and some other plants.	http://webs2.kazusa.or.jp/IntegrationDBRS/pmpj-blast/	

**Table 2:** The number of records in metabolomics-related databases at KOMICS (as of November 2013).

Database name	Number	Description
MassBase	43959	Binary raw datasets
KomicMarket	85	Biological samples, including 251 instrumental analysis datasets
	215	Chemical samples, including 488 instrumental analysis datasets
New KomicMarket temporary website	16	Number of studies, including 166 analyzed datasets
Metabolonote	34	Number of studies, including metadata for 375 instrumental analysis datasets and 765 computational analysis datasets
MS-MS Fragment Viewer	115	Analyzed flavonoids
RnR	194	Metabolite features

**Table 3:** A summary of input and output file formats and availability of sample data for preprocessing tools. The precise formats are described in the instruction manuals for each tool.

Tool name	Input	Output	Availability of sample data
PowerGet	(1) PowerGet format (text file): MSGet tool is available for generating the text files from Xcalibur raw files (2) MassBase SMS format (text file) (3) mzXML file generated from the Xcalibur raw files using the ReAdW tool^∗a^	(1) PowerGet format (text file): users can select the items and formats of the output file (2) TogoMD format (text file)	KOMICS website

FragmentAlign	*Deconvoluted peak data* (1) FragmentAlign format (text file, one of the NIST formats) (2) MassBase SMT format (text file) (3) GMD^∗b^ format (text file in NIST^∗c^ MSP format) (4).ELU file of AMDIS^∗d^ software *Chromatogram data for deconvolution* CSV file exported by Pegasus III (text file)	FragmentAlign format (text file)	KOMICS website A sample file of a standard compound library is included in the tutorial data

SpiceHit	*Electropherogram data* (1) CSV file (text file) (2) ChemStation .MS file (binary) *Standard compound library* Excel file (binary)	(1) Tab-delimited text file (2) Excel file	(1) KOMICS website (2) Included in the tool A sample of a standard compound library is included

^∗a^ReAdW tool: available at http://tools.proteomecenter.org/wiki/index.php?title=Software:ReAdW.

^∗b^GMD: Golm Metabolome Database, http://gmd.mpimp-golm.mpg.de.

^∗c^NIST: National Institute of Standards and Technology, http://www.nist.gov/.

^∗d^AMDIS: available at http://chemdata.nist.gov/mass-spc/amdis/.
