# Supplementary material for: Tools and Databases of the KOMICS Web Portal for Preprocessing, Mining, and Dissemination of Metabolomics Data
Source: Biomed Res Int. 2014 Apr 9;2014:194812. doi: 10.1155/2014/194812 (PMC4052814; doi:10.1155/2014/194812)
Supplement: Supplementary file 1 — The Supplementary Material include 3 supplementary methods and 4 supplementary figures as follows. Supplementary Method S1: The method for the evaluation of the accuracy of mass values of the peaks detected using PowerGet tool. Supplementary Method S2: The method for the evaluation of applicability of a data matrix resulting from peak alignment in FragmentAlign tool to a comparative metabolomics analysis. Supplementary Method S3. The method for the evaluation of accuracy of peak area quantified using SpiceHit tools. Supplementary Figure S1: Principal component analysis (PCA) of metabolite peaks in Arabidopsis leaves, Lotus japonicus leaves, and Arabidopsis cultured cells detected using GC-MS and aligned by means of FragmentAlign. Supplementary Figure S2: Correlation coefficients of the values of peak intensity aligned in FragmentAlign. Supplementary Figure S3: Reproducibility of peak area quantification in SpiceHit and ChemStation. Supplementary Figure S4: Comparison of values of the peak area quantified using SpiceHit and ChemStation. Four supplementary tables are as follows. Supplementary Table S1: The standard compounds used for the comparison of accuracy of mass values obtained using PowerGet and Xcalibur. Supplementary Table S2: The data used for peak alignment of GC-MS data through FragmentAlign. Supplementary Table S3: The data used for the evaluation of quantification of peaks using SpiceHit. Supplementary Table S4: Comparison of accuracy of peak area quantification in SpiceHit and in ChemStation. [file 194812.f1.pdf]

Supplementary Methods

METHOD S1: Evaluation of mass accuracy of PowerGet

The accuracy of mass values of the peaks as detected using the PowerFT module of PowerGet was compared to that estimated by the commercial software Xcalibur (Thermo Fisher) using the data from standard compounds analyzed by means of LTQ-FT (Thermo Fisher). Among the 488 analyzed datasets for 215 standard compounds we published on KomicMarket, the data obtained using ESI positive analysis coupled to LC separation were selected. When several replicates were available for the same compound, a typical one for each compound was chosen. As a result, 145 datasets were selected (Table S1).

Estimation of mass values of the compound peaks in the Xcalibur software was performed as follows: the major peak that has m/z of $[M+H]^+$ ionization correspoiding to the theoretical mass was identified as a peak derived from the standard compound.

Estimation of mass values using PowerFT was performed as follows. The mass chromatogram data in the raw file were extracted into a text file using the MSGet software. The text file was opened in PowerFT and analyzed with the default settings. Identification of the peaks from standard compounds is essentially the same as that in Xcalibur. Because a major peak from one dataset (Daidzein) was split into 2 parts at the peak's top, the "Dif. margin" parameter at the "Ion Group Setting" of the "Peak Detection" module was changed from 10 ppm to 15 ppm.

The accuracy of mass values was evaluated using the mass difference between the theoretical mass calculated from the elemental composition of the compound and the mass estimated by the software. Two peaks (Acacetin-7-rutinoside and Apigenin-7-rutinoside) were omitted because they showed mass differences greater than 5 ppm in the analysis with Xcalibur. Therefore, data from the remaining 143 compounds were used for comparison (Table S1).

METHOD S2: Evaluation of a data matrix resulting from peak alignment in FragmentAlign

Three biological sources—*Arabidopsis* leaves, *Lotus japonicus* leaves, and transgenic *Arabidopsis* cell lines (T87) transformed with a binary vector pGWB2—were used for

GC-TOF-MS analysis according to Ogawa [1]. The data obtained from 5 biological replicates for each source, namely 15 samples, were analyzed here. The raw data (the .smp file from the Pegasus III software, LECO, St. Joseph, MI) and peak data deconvoluted using Pegasus III in text format (.mst file) were obtained from MassBase. The IDs of the data files are shown in Table S2.

The peak data deconvoluted by means of Pegasus III software in .mst files were imported into FragmentAlign, and peak alignment was performed with default settings. The step of prealignment (to find the internal standard peaks for fine matching of retention times) was omitted. The alignment results were saved in a text file without any manual curation.

The data for metabolite peaks, which were detected in at least 3 of the 15 samples, were used for PCA. The linear value of peak intensity was transformed to the log scale with base 10. The missing values were filled by a small value, which was 1/10 of the minimum intensity of the peaks among the 15 samples as a tentative background. PCA was performed in the R software (http://www.r-project.org/, version 3.0.1) using the prcomp package without scaling.

Pearson's correlation coefficients of the values of peak intensity between 2 replicate samples were calculated using the CORREL function of Excel (Microsoft Corporation) using all peak data detected in each sample.

METHOD S3: CE-MS analysis

We prepared 2 types of samples for the evaluation: mixtures of amino acids as authentic compound samples, and *Arabidopsis* cell extracts as biological samples. As authentic compounds, a series of amino acid mixtures which contained 10, 50, 100, and 1000 μM of each amino acid (Gly, Ser, Pro, Val, Thr, Cys, Ile, Leu, Asn, Asp, Lys, Glu, Met, His, Phe, Arg, Tyr, Trp, and cystine) in Milli-Q water were prepared. As biological samples, soluble extracts from *Arabidopsis thaliana* suspension-cultured T87 cells [2] were prepared. This cell line was obtained from RIKEN BioResource Center (Tsukuba, Japan) and maintained as described by Ogawa [1]. The cells after 3, 10, and 14 days after subculturing were collected on filter paper, washed once with distilled water, immediately frozen in liquid nitrogen, and stored at -80°C until use. Extraction of metabolites from the cells and pretreatment were performed as described previously [3]. As an internal standard chemical, methionine sulfone was added to all the samples. The capillary electrophoresis, positive mode detection with the electrospray ionization, was

performed on an Agilent CE-Capillary Electrophoresis System as described by Urano [3]. The m/z values of the positively ionized amino acids and of the internal standard were set for the SIM scan. Triplicate and single sample injections were performed for each concentration of an amino acid mixture and for each biological sample, respectively. Chemicals were purchased from Sigma-Aldrich Co. (Tokyo, Japan).

In the data analysis using ChemStation, peak areas were calculated by means of the integration function of the software. The parameters for automatic peak detection were set as follows: Slope Sensitivity 5000, Peak Width 0.07, Area Reject 100, Height Reject 100, and Shoulders, OFF. The automatically detected peaks were refined using the manual integration functions, and the resulting migration time and peak area were transcribed manually into Excel worksheets (Microsoft Corp.). Amino acid peaks in the biological samples were identified using manual calculation of the relative migration times and comparison with those of the authentic samples. In the analyses in SpiceHit, a standard compound library was constructed with the data from authentic samples, and it was used for identification of peaks in the biological samples. Default parameters were used for peak detection and identification.

Supplementary Figures

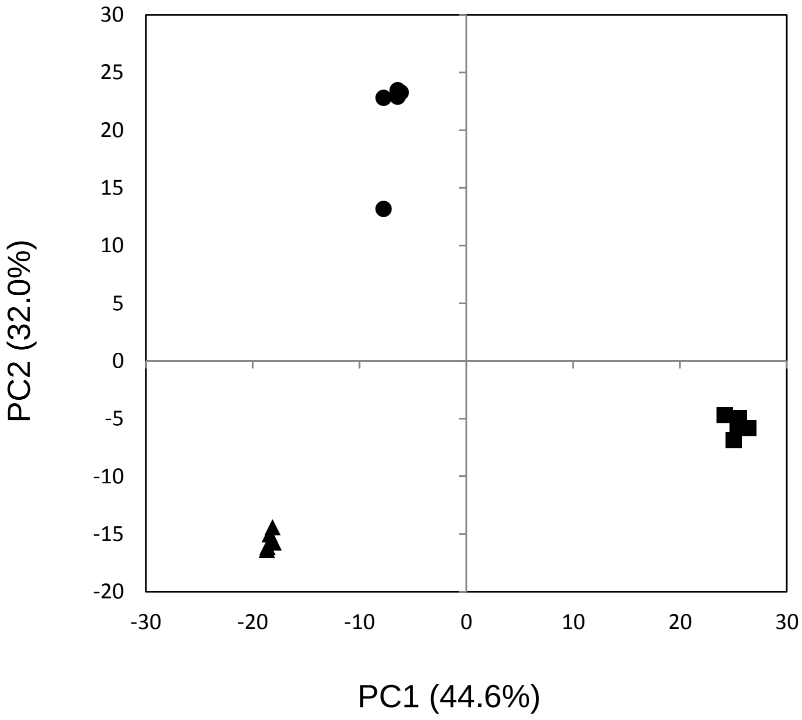

FIGURE S1: Principal component analysis (PCA) of metabolite peaks in *Arabidopsis* leaves (circles), *Lotus japonicus* leaves (squares), and *Arabidopsis* cultured cells (triangles) detected using GC-MS and aligned by means of FragmentAlign.

(a) Arabidopsis leaf

	MDGC1_01990	MDGC1_01992	MDGC1_01996	MDGC1_01998	MDGC1_02000
MDGC1_01990	–	0.800	0.822	0.781	0.742
MDGC1_01992		–	0.909	0.862	0.914
MDGC1_01996			–	0.919	0.913
MDGC1_01998				–	0.878
MDGC1_02000					–

minimum	0.742
maximum	0.919
mean	0.854

(b) Lotus japonicus leaf

	MDGC1_01980	MDGC1_01982	MDGC1_01984	MDGC1_01986	MDGC1_01988
MDGC1_01980	–	0.961	0.956	0.948	0.967
MDGC1_01982		–	0.956	0.956	0.972
MDGC1_01984			–	0.948	0.946
MDGC1_01986				–	0.967
MDGC1_01988					–

minimum	0.946
maximum	0.972
mean	0.958

(c) Arabidopsis cultured cell

	MDGC1_02742	MDGC1_02744	MDGC1_02746	MDGC1_02748	MDGC1_02750
MDGC1_02742	–	0.928	0.879	0.930	0.940
MDGC1_02744		–	0.826	0.944	0.892
MDGC1_02746			–	0.901	0.849
MDGC1_02748				–	0.931
MDGC1_02750					–

minimum	0.826
maximum	0.944
mean	0.902

FIGURE S2: Correlation coefficients of the values of peak intensity aligned in FragmentAlign.

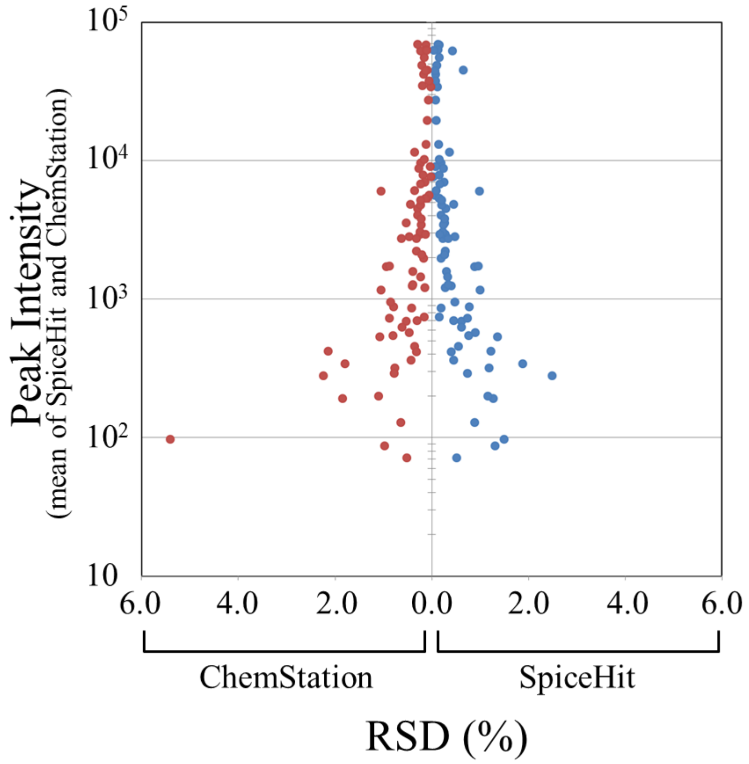

FIGURE S3: Reproducibility of peak area quantification in SpiceHit and ChemStation. The relative standard deviation (RSD = standard deviation / mean) of an amino acid peak was calculated using triplicate analysis of amino acid solutions. The mean values of peak area for the triplicates were estimated using SpiceHit and ChemStation and were further averaged to show the extent of the peak intensity (the vertical axis). The peak area was transformed to a logarithm with base 10 for the calculation of mean values and RSDs. Four solutions containing a mixture of amino acids at 10, 50, 100, and 1000 μM were analyzed in triplicate, and all the identified amino acid peaks were plotted.

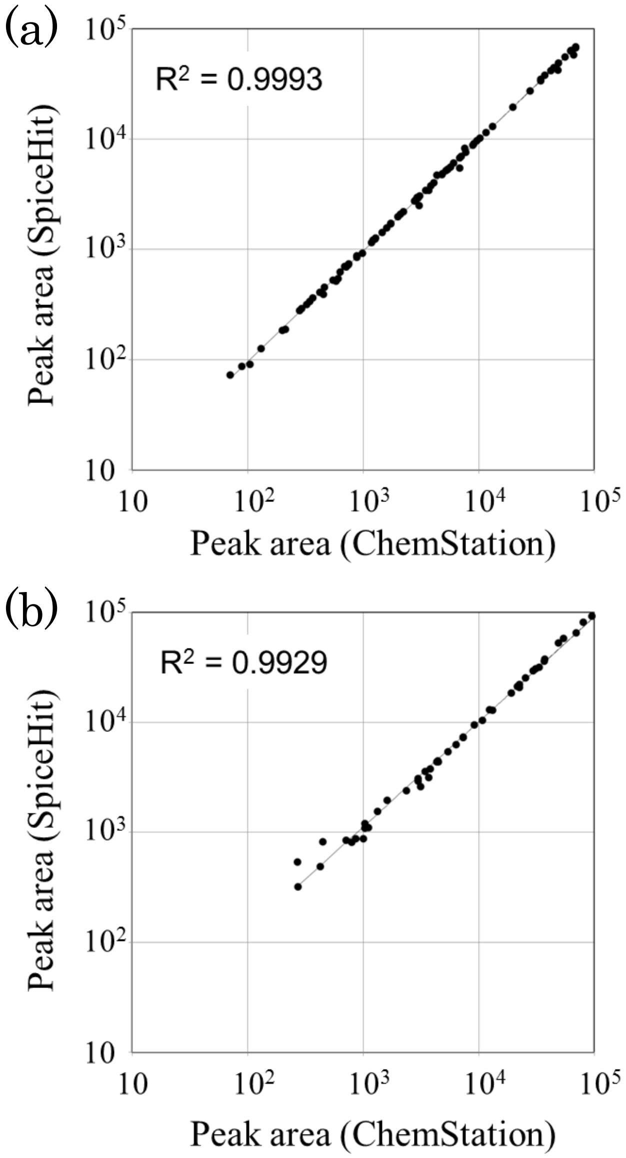

FIGURE S4: Comparison of values of the peak area quantified using SpiceHit and ChemStation. (a) Amino acid peaks in the amino acid solutions. The mean values of the peak area detected in the triplicate analyses of 4 concentrations of amino acid solutions (10, 50, 100, and 1000 μM) were plotted. The peak area was transformed to a logarithm with base 10 for calculation of the mean value. (b) Amino acid peaks in the extracts of *Arabidopsis thaliana* suspension-cultured T87 cells. A single analysis was performed for each cell sample collected 3, 10, and 14 days after initiation of subculturing.

References

[1] Y. Ogawa, H. Suzuki, N. Sakurai et al., “Cryopreservation and metabolic profiling analysis of Arabidopsis T87 suspension-cultured cells,” *Cryo letters*, vol. 29, no. 5, pp. 427-436, 2008.

[2] M. Axelos, C. Curie, L. Mazzolini et al., “A protocol for transient gene expression in *Arabidopsis thaliana* protoplasts isolated from cell suspension cultures,” *Plant Physiol Biochem*, vol. 30, no. 1, pp. 123-128, 1992.

[3] K. Urano, K. Maruyama, Y. Ogata et al., “Characterization of the ABA-regulated global responses to dehydration in Arabidopsis by metabolomics,” *Plant J*, vol. 57, no. 6, pp. 1065-1078, 2009.
